# Involving trained community health mediators in COVID-19 prevention measures. A process evaluation from Bremen, Germany

**DOI:** 10.3389/fdgth.2023.1266684

**Published:** 2023-10-11

**Authors:** Tilman Brand, Marieke Gerstmann, Florence Samkange-Zeeb, Hajo Zeeb

**Affiliations:** ^1^ Department of Prevention and Evaluation, Leibniz Institute for Prevention Research and Epidemiology (LG), Bremen, Germany; ^2^ Department of Prevention and Evaluation, University of Bremen, Bremen, Germany

**Keywords:** migration, process evaluation, participation, health mediators, COVID-19

## Abstract

**Objective:**

The objective was to assess the feasibility of incorporating trained community health mediators in COVID-19 prevention in a multicultural and disadvantaged setting in Bremen, Germany. Specifically, we aimed to develop and implement measures corresponding to the needs of the residents and to analyse the role of digital communication tools and sustainability factors of the health mediator approach.

**Methods:**

A comprehensive process evaluation using 41 qualitative interviews with residents, mediator short surveys and group discussions, work documentation sheets, and a stakeholder workshop was carried out.

**Results:**

Uncertainties due to changing regulations, a lack of trust and fear of potential side effects were major themes identified in the needs assessment. The eight mediators documented more than 1,600 contacts. Digital communication via Facebook was a useful tool, but personal contacts remained crucial for communicating with residents. The participatory approach, multilingualism and the flexibility to react to dynamic situations were identified as relevant factors for the success and sustainability of the health mediator approach.

**Conclusion:**

Multilingual health mediators can facilitate contact with and dissemination of health information to different communities and also can play an important role in pandemic preparedness.

## Introduction

Socioeconomic, ethnic and geographical inequalities in COVID-19 infections and mortality have been found in many countries worldwide (1–7). For example, in the UK mortality rates in deprived areas were more than twice as high as in affluent areas (8, 9). In Germany, differences in the distribution and severity of COVID-19 across social strata were observed (10–12). The reasons for the observed social disparities are multifaceted, including working and living conditions (6, 13). Poor working or living conditions give people less opportunities to protect themselves from a COVID-19 infection (14, 15). Additionally, for people working under poor conditions some of the prevention measures (e.g., staying in quarantine) were accompanied by an existential fear to lose income and, eventually, to lose the job (16).

Vaccination was one of the most important strategies to control the COVID-19 pandemic (17, 18). In a study conducted in 2021, the Robert Koch Institute observed that while German people without a migration background (i.e., defined as being born abroad or having at least one parent who was born abroad) had a slightly higher vaccination rate than those with a migration background, the willingness to get vaccinated was higher among the unvaccinated people with a migration background (19). The explanations given for the lower vaccination rate among people with migration background included language barriers, discrimination experiences in the health sector, as well as general socioeconomic factors such as education and income. Language however seemed to be the biggest factor regarding vaccination decision. The better the German language skills, the higher the vaccination rate (19).

In earlier pandemics such as Ebola, H1N1 and Zika, one important element in fighting the pandemic was community involvement (20). The term community can be defined as “a group of people with diverse characteristics who are linked by social ties, share common perspectives, and engage in joint action in geographical locations or settings” (21). The community involvement approach was used to overcome suspicion regarding the existence of the disease and mistrust in the government. Activities implemented in earlier pandemics included involving religious leaders, building partnerships with the communities and formulating key arguments for behavioural change. During the COVID-19 pandemic, some international agencies raised concern that communities were not being sufficiently involved. In general, there are hardly any reports from high-income countries on community involvement during outbreaks (20).

Health mediators are lay persons who receive lower levels of education and training than professional healthcare providers such as nurses and doctors or professional health educators. They can translate and deliver health information and advice in a culturally appropriate manner and establish trustful relationships with population groups which are otherwise considered as hard to reach. Since the 1990s, there has been an increase in the use of health mediators to help improve accessibility and quality of health care for refugees and migrants (22–24). The role of the mediators is to improve communication and understanding between residents and health-care providers by reducing linguistic and sociocultural differences (25). Their work includes informal mediation with family and friends to help empower them so as to prevent and reduce conflicts between residents and providers (22). The health mediator approach resembles the community health worker concept which has been deployed in low and middle income countries (LMICS) and some high income countries (HIC, especially in the US) for quite some time (26). One of the differences between the approaches is that health mediators do not deliver diagnostic services or provide curative care. Rather, they focus on reducing language barriers and providing psychosocial support.

The objective of this study was to assess the feasibility of involving trained health mediators in COVID-19 prevention measures in a multicultural and socioeconomically disadvantaged neighbourhood in Bremen, Germany. More specifically, we aimed (a) to explore the needs and concerns of residents regarding COVID-19 prevention measures, including vaccination uptake, (b) to develop and implement measures that correspond to the needs of the residents, (c) to assess the role digital communication tools played in the work of the mediators, and (d) to identify factors that are relevant for the sustainability of the mediator approach.

## Methods

In the context of a project than was implemented between July 2021 and June 2022, we carried out a comprehensive process evaluation using several data sources including qualitative interviews with residents, mediator short surveys and group discussions, work documentation sheets, and a stakeholder workshop.

Participatory action research was the underlying methodology of this study (27). That means that the researchers teamed up with multilingual lay health mediators and engaged in a reflective cycle of collecting and analysing data and then deciding upon actions.

Ethical approval for the study was obtained from the ethics committee of the University Bremen, Germany (reference number 2021-07). All participants provided written informed consent. The COREQ-Checklist was used as a guideline for reporting the qualitative data (28).

### Study team

The research was conducted by a team of four scientists from the Leibniz Institute for Prevention and Epidemiology-BIPS (MG, TB, FSZ, HZ) together with eight mediators, who were employed by the institute initially for the duration of the project. All research team members had previously worked in projects conducted in disadvantaged neighbourhoods, and the scientists had experience working with community researchers.

### Study setting

The study was conducted in the city district of Osterholz in Bremen, home to approximately 37,000 people (Bremen: ca. 567,000). The neighbourhood was selected as it is one of Bremen's most culturally diverse city districts, with 53.5% of the population having a migration background. The average annual income in the neighbourhood is lower than that of the overall city (24,500 vs. 33,000 Euro) (29). According to health statistics from 2020, the COVID-19 infection rate in Osterholz was 20.5 per 1,000, three times higher than in high-income city districts and the highest in Bremen at that time (30).

The whole project was situated in the Leibniz Living Lab (LLL), a BIPS community office situated in Osterholz (31). The LLL served as the project hub where the project team worked and interacted with mediators and stakeholders.

### Recruitment and training of mediators

The eight mediators were recruited through local partners and pre-existing networks from earlier research activities in the neighbourhood. The requirements for becoming a health mediator were having a large social network in Osterholz, and good language skills in in German and another language spoken in the neighbourhood. The hired mediators were all female, aged 24–50 and spoke the following languages: German, English, Twi, Tamil, Turkish, Arabic, Russian, and Macedonian. Most of the mediators worked in centres where families (parents, children grandparents) meet to get to know each other and share ideas/experiences, so-called *Mütterzentren* in Germany. These mediators hence had regular close contact to residents.

The mediators received continuous training during the course of the project. The training sessions were aligned to the different phases of the project and covered various topics including recruitment of participants and conducting interviews, ethical and data protection aspects, interpersonal communication and the use of Facebook, basic information on the COVID-19 virus and its transmission, vaccines, and fake news. Some of the sessions were scheduled, while others took place on demand and covered topics raised by the mediators.

### Cooperation with other stakeholders

Throughout the study, the collaboration with social service managers working in the neighbourhood, was intensified. This concerned in particular cooperation with professional health educators employed by the Federal State Association for Health and the Academy for Social Services, Lower Saxony (LVG & AFS Nds e.V.) and Federal State Association for Health in Bremen (LVG Bremen), with funds provided by the federal state of Bremen. The professional health educators were employed during the peak of the pandemic and their role was to provide COVID-19-related information to citizens in disadvantaged neighbourhoods (32).

### Needs assessment

The mediators carried out semi-structured interviews focussing on COVID-19 related needs and concerns among residents in Osterholz using an interview guide they had helped develop and had pre-tested. The interview centred on COVID-19-related knowledge, vaccination, rules and the effects of the pandemic on everyday life (see Supplementary File 1).

Interviews were conducted with individuals who lived in the city district and were at least 18 years of age. The mediators used their personal networks to identify and recruit interview partners from diverse micro-communities. The interviewees determined the interview location. Some interviews were conducted at the interviewee's home, and others at their workplace or over the phone.

The interviews were conducted during the phase when severe contact restrictions were in place, and the COVID-19 delta variant was dominant. There was no mandatory testing for people who had been vaccinated twice, and infected persons had to quarantine for 14 days (19, 33). At that time, slightly more than three-quarters of the population in Germany had already been vaccinated (34).

The study information, consent form and the interview guide were translated into the mediators' main languages.

### Development and implementation of activities to support COVID-19 prevention measures

The needs assessment was followed by the development and implementation phase of COVID-19 prevention measures. As personal contacts were reduced to a minimum during this phase, the mediators were equipped with smartphones on which they could be reached by the members of the respective communities for advice and assistance regarding COVID-19 related issues. The smartphones hence served as a communication and a dissemination tool.

Information on the availability of the mediators and their role was distributed via project flyers and posters at community centres and public places. Furthermore, to enhance project dissemination to a wider audience and to facilitate the availability of new COVID-19 regulations and vaccination opportunities in different languages, each mediator created Facebook profiles. The latter were created exclusively for the project and the mediators used these to promote short video messages in Arabic, Russian, and Turkish they had created via Facebook advertisement. The advertisement posts were tailored to residents in Osterholz. Facebook was chosen because the needs assessment showed that this was the most relevant source of information among the social media platforms used by the communities concerned (next to WhatsApp). It was also one of the sources that was most often associated with fake news.

In line with the participatory approach, the project coordinator (MG) held regular meetings with the mediators during the development and implementation phase of the project. The meetings served as platforms to reflect about the project in general, generate new ideas as a team, as well as for continuous planning and providing background support.

The main tasks of the mediators in this phase were individual counselling and serving as contact points for residents, as well as supporting and promoting the local vaccination campaign. Further activities that were implemented based on the needs assessment results were for example: developing and disseminating own COVID-19 educational material (also in form of Facebook videos), a painting workshop with children, where the focus was on COVID-19 vaccination, and a stakeholder workshop on vaccination of children.

### Data collection for the process evaluation

To gain a rapid overview of the current situation on the needs of the residents, barriers, reach and impact of the mediators' work, the mediators completed a short survey (anonymously) at month 2 (September 2021) and month 10 (May 2022).

In addition, the mediators anonymously documented all queries and issues raised by the community members using a pre-set documentation sheet that was collected by the project coordinator every two weeks. The sheet included information on the number and type of contacts the mediators had had, whether these had been face-to-face or digital, as well as the topics covered and material used.

Moreover, at the end of the project, all mediators participated in a discussion round during which they reflected on different aspects of the project, including the project work itself, the training sessions, support and communication as well as on their empowerment and the level of participation throughout the project. In a final stakeholder workshop, the concept and sustainability factors of the mediator approach were discussed with stakeholders, such as local policy makers, health educators and residents.

### Data analysis

The semi-structured interviews for the needs assessment were audiotaped and transcribed verbatim by a professional multilingual transcription service. The latter also translated transcripts that were not in German or English into German. The meaning of sections that were found to be unclear were cross-checked with the mediators. After transcription, the data were anonymised and analysed according to applied thematic analysis (35) using MAXQDA. The codebook was created by MG and FSZ. They independently coded the same transcripts using the interview guide as a basis, and then met to discuss their output and to agree on a basic structure. The coding of the transcripts was then done by MG in consultation with FSZ and TB. The final version of the codebook had four main topics: COVID information (e.g., sources of information used, barriers in the information seeking process, gaps, fake news), COVID rules (reasons for and against compliance with COVID rules), COVID vaccination (attitude towards COVID vaccination) and COVID impact on everyday life (e.g., social and economic consequences of the pandemic), which were divided into different themes (see Supplementary File 2 and 3). The interview results were discussed with the mediators in the context of a joint evaluation, and the output formed the basis for the intervention development.

The short surveys conducted among the mediators and the documentation sheets were analysed descriptively using MS Excel. The mediators recorded each time they contacted someone within the context of the project, without indicating whether or not they had contacted the person before.

Furthermore, to evaluate the Facebook video performances, key figures available on Meta Business Suite were used. The ensuing results were discussed with the mediators and they contextualized and amended them based on their experiences in the project.

Finally, the main project results, including strengths and weaknesses, as well as factors related to the sustainability of the mediators were discussed with local stakeholders. Factors contributing to the sustainability of the mediator approach were extracted from group discussion notes and workshop documentation. Plain language summaries of the findings in the respective languages were disseminated to the interview participants.

## Results

### Study population characteristics

Overall, 41 interviews were conducted in August and September 2021. Most of the interview participants were known to the mediators beforehand. Of the 41 interviews, 18 were conducted in German, 12 in Arabic, four in Twi, three in Turkish, three in Russian and one in Tamil. On average, the interviews were 24 min long (range 8–70 min) (Table 1).

**Table 1:** Characteristics of the needs assessment participants (*n* = 41).

** **	Frequency
Gender	
Men	7
Women	33
Diverse	–
*Not reported*	1
Age	
Average age	43 years
Age range	19–67 years
Employment status	
Students	8
Pensioners	4
Housewives	5
Employed	19
Unemployed	4
*Not reported*	1
Education (highest level completed)	
Academic degree	3
Training degree	14
No degree	16
*Not reported*	8

### Residents' needs and concerns during the COVID-19 pandemic

In terms of COVID information many respondents felt well informed and used a variety of information sources. However, they also pointed out that most of the information was only available in German and thus identified the need for multilingual COVID information. In addition, the respondents mentioned people with limited reading and writing skills and older adults as being particularly at risk for receiving no or misinformation. Facebook and WhatsApp were widely used among the respondents, but they were also concerned that these sources were drivers of misinformation and fake news. In general, the respondents perceived webpages from known institutions as being more trustworthy than social media channels. Several respondents were not satisfied with the amount of information they received from public institutions such as the local government, schools, and the public health office. Others described that ever-changing regulations and information overload made them ignore COVID related information. They called for someone to filter the information and provide personalized advice for them.

Apart from situations in which adhering to COVID rules such as wearing a face mask or social distancing was difficult, respondents assumed that not believing in COVID, tiredness after several years of the COVID pandemic and the belief that being vaccinated would protect them from an infection were the main reasons for people not to follow the COVID rules. On the one hand, the respondents identified the need for more information and the need for credible explanations of why the rules are essential. On the other hand, several respondents called for stricter enforcement of the rules.

With regard to COVID vaccination, a mixture of uncertainty and fear was expressed. When asked about the reasons against vaccination of children, one respondent answered:

*“Well, because they think it hasn't been researched enough and it's unsafe, that they're not being properly educated about how safe it is by now. Well, they're assuming that other testing of drugs or viruses or vaccines took longer periods of time and now this had to be done in a very short period of time.” (female, 61 years)*

The word *fear* itself in connection with the pandemic was used in a total of 161 times and in three quarters of the interviews (31/41). It was mentioned not only in relation to the vaccination but also in relation to general infection, infecting family and friends, long-term consequences, correct behaviour in public, and quarantine regulations. As one interviewee put it:

*“We almost didn't even dare to say “hello” to our neighbours when we ran into them in the stairwell.” (female, 59 years)*

**Figure 1:**
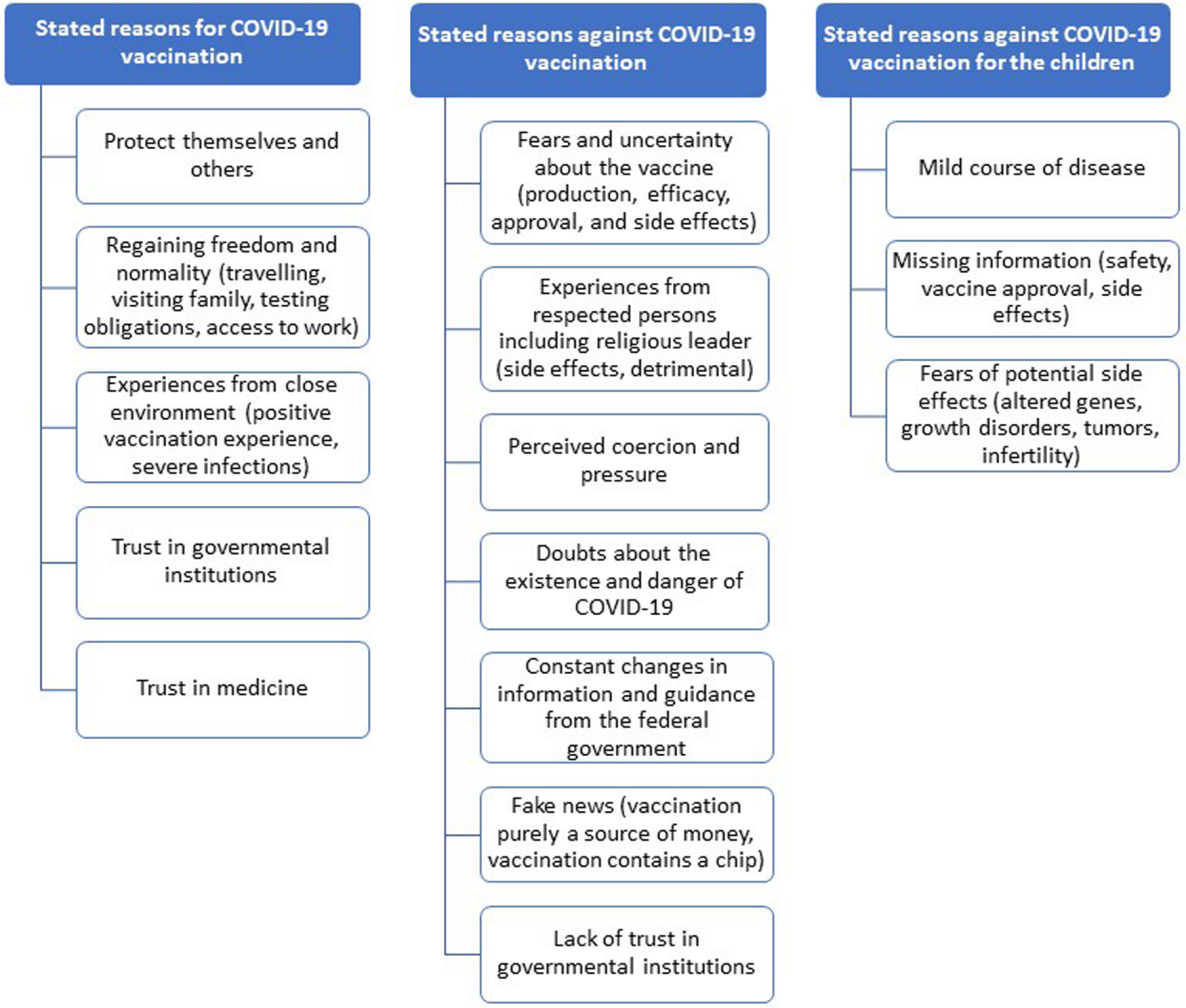
Stated reasons for and against COVID-19 vaccinations form participants of the needs assessment (*n* = 41; source: own figure).

The reasons given by the respondents regarding whether or not they had been vaccinated against COVID-19 various ranged from the wish to protect others (pro vaccination) to lack of trust in government institutions (contra vaccination) (Figure 1). As shown in the following statement, the opinion and behaviour of family and friends also played a big role in the decision-making:

*“What made me want to get vaccinated was the fact that my relatives and friends had already been vaccinated. At first, I was against getting vaccinated, but they assured me that there was nothing wrong with it because they themselves had already been vaccinated” (female, 40 years.)*

The social and economic consequences of the pandemic were not only negative. Some respondents mentioned reduced costs for mobility, reduced working hours, and an increased recognition of the value of social contacts as positive aspects. Nevertheless, many respondents were burdened by limited social contacts or feeling uncomfortable with social contacts, and suffered financial strains. Homeschooling and reduced childcare hours were identified as the biggest challenges for families. The need for hands-on psychosocial support through low-threshold neighborhood and mediation services was mentioned several times.

### Needs and concerns addressed by the multilingual trained health mediators

Needs and concerns that were considered as being important for the practical implementation of the mediator approach were: reducing fears and knowledge gaps through the dissemination of multilingual COVID-19 information, addressing vaccination of children as a particularly sensitive topic, the importance of a personal approach and the role of the social environment regarding confidence in preventive measures.

According to the documentation forms submitted by the eight mediators, they jointly had approximately 1,600 contacts with residents during the course of the project, mostly via telephone, personal contact or e-mail/short messages (Table 2). The majority of the people contacted were aged 19–64 years (90%), more than three-quarters were female (77%), and in 50% of the cases, the contact was in German.

**Table 2:** Description and documentation of health mediator contacts with residents.

Type of contact (frequencies)	
Personal	559
Via telephone	838
Via emails/messages	537
Via social media posts	178
Via group meetings (incl. digital)	187
Characteristics of contacted residents	
Age (%)	
0–18 years old	6.8
19–64 years old	89.5
65 + years old	6.4
Gender (%)	
Women	77.3
Men	23.2
Diverse	0.1
Contact language (%)	
German	48.7
Foreign language	51.3
Topics (multiple answers possible; %)	
Regulations	70.0
Vaccination	58.8
Testing	13.8
COVID-19-disease	3.8

The topic most frequently addressed during the contacts with residents was current COVID-19 regulations, followed by vaccination. Other topics included testing, proper behaviour after a positive test, self-help in the event of COVID-19 infection, and protecting at-risk groups. In the course of the project, concern was also raised in relation to Ukrainian refugees, for instance, concerning availability of COVID-19-vaccination and rapid test opportunities for them.

The mediators also frequently used multilingual flyers to disseminate information on COVID-19.

### Use of digital tools for communication within the project

All three Facebook videos achieved a large reach in relation to the size of the district (see Table 3). The duration of the videos ranged from 29 s (Arabic), to 101 s (Russian). The Arabic video had the largest reach with 1,185 user views, followed by the Russian video (1,063 user views). While the Turkish video had the least number of user views (866), the proportion of plays (views) longer than 15 s was more than double that of the Russian video views (87% vs. 36%). The number of the Arabic video views longer than 15 s on the other hand was higher than the number of user views themselves, indicating multiple plays by one user.

**Table 3:** Facebook videos reach.

** **	Video in Arabic	Video in Russian	Video in Turkish
Reach (count Facebook user)	1,185	1,063	866
Plays longer than 15 s^a^	1,300	378	757
Viewer main age	25–34	25–64	45–54
Viewer main gender	Men	Women	Men

^a^ Can include multiple plays by one user.

### Factors that affect the implementation and sustainability of the mediator approach

During the group discussions with the mediators as well as in the final stakeholder workshop, the participants expressed great satisfaction with the project's output. The mediators and researchers also found the participatory approach to be very instructive and enriching. While the mediators felt very self-efficacious in their work and strengthened by their participation in the project, they also reported difficulties in maintaining the balance between protection of own privacy and the project aim to be a visible contact person in the neighborhood. Some of the mediators feared that they could become targets of verbal or physical attacks from critics of the COVID-19 regulations. Another challenge was the blurring line between being a mediator and other social roles such as being a friend or neighbour.

The success factors identified for the implementation of the approach were the flexible response to changing conditions during the pandemic and other crises such as the Ukrainian war, as well as the mediators' great freedom to make decisions and participate in shaping the project. The contribution of the mediators was seen primarily in the trust-centred and low-threshold approach to people who were uncertain about COVID-19 regulations and vaccinations. The multilingualism in the team was particularly beneficial. The factors identified for the sustainable implementation of the approach were the collaboration with independent and well-known institutions in the district, and intensive, continuous background support for the mediators.

### Mediator short survey

The short survey about the current COVID-19 situation in the social network of mediators showed that current regulations and guidelines on COVID-19 played less of a role in May 2022 compared to September 2021, but questions on the future were raised more and more frequently. Furthermore, the mediators' own assessment of the impact of their work towards improved pandemic management shifted from being fairly positive to positive in the second survey.

## Discussion

This study assessed the feasibility of involving trained community health mediators in COVID-19 prevention measures in a multicultural and socioeconomically disadvantaged urban setting. In the process, different activities were developed corresponding to the needs and concerns about COVID-19 prevention measures among residents living in this community. The work with the mediators proved to be feasible, with many residents being reached and different activities being well implemented. The mediators and the research team also benefitted from the participatory approach applied and the close collaboration achieved.

This study responded to the call for a stronger community involvement in efforts to address the COVID-19 pandemic, especially in high-income countries (20). Through the collaboration with the mediators, contact was made with otherwise underserved population groups. The approach also enabled the project to tailor COVID-19-related information to the needs of the said populations and deliver the information in a culturally appropriate manner, which is one of the core aims of health mediator approaches (22). The reasons against vaccination during the COVID-19 pandemic that were given in our study (e.g., mistrust in the government) are very similar to those expressed during the 2014–2015 Ebola epidemic in a totally different context (20). Our study findings indicate the need for measures to address and reduce fears and misconceptions during pandemics, and to fill knowledge gaps.

Similar to other studies (36–39), we found that social media played a dual role during the pandemic. On the one hand, our interview respondents recognized that social media added to the information overload and spread of misinformation and fake news. On the other hand, the social media activities carried out by the health mediators proved to be a promising way to reach population groups that are unlikely to be reached via other information channels such as leaflets, German-language newspapers, or German-language television. However, personal contact with community residents remains crucial.

Through a critical lens health mediator approaches have been depicted as neo-colonial tools of epistemic violence as they portray the communities that the mediators represent as “the problematic others” (40). We tried to avoid the risk of epistemic violence by following the principles of participatory action research which meant that the research team and the mediators agreed on topics and methods during the project lifetime.

### Practical implications

Although the potential for upscaling and sustainability is hard to assess from this study, discussions with local stakeholders and the health mediators provided at least some qualitative insights. In the context of community health worker programs, Zulu et al. (41) highlighted the importance of several health system elements concerning the integration of such programs into national health systems. Among these are governance and leadership, financial resources, human resources, and population embeddedness. While an integration into the management structure of the public health offices would increase the scalability and sustainability of the health mediator approach, the local stakeholders and the health mediators emphasized the role of trustworthy local nongovernmental organizations and the cooperation with an independent research institute for achieving community acceptance and embeddedness. In this project, the mediators were hired as salaried members of BIPS, which was highly valued by the mediators. Nevertheless, this should not conceal the fact that health mediation is a marginal and, in many cases, precarious employment form and not a full-time job, as pointed out by Verrept (22). As a consequence, fluctuations among the mediators are quite common (26). From the literature on community health workers, there is also evidence that higher-educated community workers are more likely to drop out, although their work was found to be more effective (42). In any case, a high fluctuation needs to be planned for when scaling up a mediator program. Similar to the findings from our study, the literature on community health workers and health mediators also confirms that strong and ongoing supervisory support is essential in order to achieve a high quality of work and to increase the motivation and satisfaction of the mediators.

### Strengths and limitations

The main strength of the study is that it made use of a participatory approach, whereby the mediators were involved in all stages of the project as equal partners, and they in turn collaborated with the residents they contacted. Throughout the project, the mediators continuously developed a sense of ownership and belonging that showed itself in the way they conducted their work.

The greatest contribution of the mediator approach probably lies in the relationships they managed to build with the individuals they reached and the positive experiences made. These can be used as a resource, for instance, for pandemic preparedness.

One limitation of the study is that, due to its design, the exact impact of the mediators on the vaccination rate could not be determined. Nevertheless, it should be noted that the basic immunization rate in Bremen was rather high (87.5% in Bremen vs. 76.4% nationwide) (34). The mediators may have contributed to this through their work supporting safe vaccination decisions, passing on information about vaccination opportunities in the community, and listening and acting on queries from the residents.

Regarding generalisability, it is difficult to say whether the approach would work in other neighbourhoods or cities in the same way as in the specific setting. However, many urban communities in Germany and elsewhere share social and economic characteristics of Osterholz.

The study was conducted during the period where in-person events and contacts were restricted by COVID-19 regulations, which is an obstacle for participatory research (43). Nevertheless, the feedback from the mediators gave us the impression that the participatory approach worked well despite and in view of these restrictions.

## Conclusion

Co-working with community health mediators in a socio-economically deprived city district on COVID-19-prevention in participatory manner was feasible and led to a large number of personal contacts between mediators and residents. We believe that health mediators can be an important resource for pandemic response and preparedness as they can develop trustful relationships in communities that may otherwise not be sufficiently reached by public communication strategies.

## Data Availability

The raw data supporting the conclusions of this article will be made available by the authors, without undue reservation.
