# Supplementary material for: Involving trained community health mediators in COVID-19 prevention measures. A process evaluation from Bremen, Germany
Source: Front Digit Health. 2023 Oct 11;5:1266684. doi: 10.3389/fdgth.2023.1266684 (PMC10598750; doi:10.3389/fdgth.2023.1266684)
Supplement: Supplementary file 1 [file Table1.docx]

**Interview Questions**

Introductory: I would like to hear from you about your perspective on the Corona pandemic, i.e., how well informed you and the people you deal with feel, where you get information, what concerns you have, and so on. Feel free to tell us from your own perspective and also about impressions from your neighborhood or district.

First, I would like to ask a few questions about Corona information in general:

- First, how well informed are you about Corona - especially about the ways Corona is transmitted and things people can do to avoid infecting themselves and others?
- What do you think: How well informed are other people you know about Corona?

If not well:

- - What do you think is the reason?
  - What could be done to improve the information situation?

If good:

- - How do you inform yourself about Corona?
  - How do other people you know inform themselves?
- What role do the Internet and social media (e.g. Facebook, WhatsApp, or Instagram) play for you and the people you know in regard to spreading Corona information?

Now I would like to talk about Corona rules and Corona vaccinations:

- How well would you say you follow the Corona rules, such as keeping distance, hygiene, and wearing a face mask?
- How about the other people you know? How well do they follow Corona rules?

If not well:

- - What makes it difficult to stick to the Corona rules?
  - What kind of support do people need to adhere to the Corona rules?
- What do you think: how well do other people know about what to do if they were tested positive for Corona?
- What do you think: How well do people know how to behave if they have had contact with a person who was tested positive for Corona?
- Have you already been vaccinated against Corona?
- If yes: What convinced you to get vaccinated?
- If no: Why not?
- If you have children, would you get your children vaccinated?
  - If yes: What is the reason for getting your children vaccinated?
  - If no: Why not?
- How would you rate the willingness of the other people you deal with to get vaccinated?
- If you know people who don't want to be vaccinated: What do they say about it?
- What is needed for people to make a safe decision to get vaccinated or not?

And now I would like to ask questions about how you are dealing in your everyday life with Corona:

- For many people, life has changed a lot because of the Corona pandemic - what about you?
  - How is it for other people you deal with?
- Where do you and the people you deal with get support in everyday life during the Corona pandemic? (For example, going to the doctor, going to the public authority offices...).
  - What kind of support would you like to have?
  - How exactly should these support services look like?
- How good is the communication about Corona from the social/health authorities in your district (for example: doctor, social services, kindergarten, employer, university)?
  - How could the communication be improved?
- Is there anything else related to the Corona pandemic that you would like to address within this interview?

Finally, I would like to ask you questions about yourself:

- How old are you?
- How would you describe your gender?
- In which country were you born?
- What is your native language?
- How many people -excluding yourself- do you live with?
- To which group would you count yourself?
  - Employed or self-employed
  - Retired
  - Pupil or student
  - Unemployed or permanently sick
  - Fulfilling domestic tasks
  - Other....
- What is your highest professional education?
  - No professional training
  - Professional training / apprenticeship
  - University degree
  - Doctorate
