# Supplementary material for: Involving trained community health mediators in COVID-19 prevention measures. A process evaluation from Bremen, Germany
Source: Front Digit Health. 2023 Oct 11;5:1266684. doi: 10.3389/fdgth.2023.1266684 (PMC10598750; doi:10.3389/fdgth.2023.1266684)
Supplement: Supplementary file 2 [file Table2.docx]

Codebook- shortened

| **Main Codes** | **Memo** | **Frequency** | ***Example*** |
| --- | --- | --- | --- |
| **COVID Information** |  | **613** |  |
| Information Situation | Describing how the information situation is perceived, including barriers to information, Fake News, amount of information | 105 | *„And somehow it was always just like a continuous loop of the same thing and you didn't have the feeling that new information was coming. Or it was only reported about the high numbers and somehow not really about what has to be done and how to act. And then you always have the problem with news, fake news, or that you have false information, everyone reports something different.”(female, 21 y.)* |
| Source | including positive and negative perception of the stated sources (workplace, educational institutions, social surrounding, health institutions, television radio, newspaper, news unspecified). Soruce of informationof the vaccination is coded COVID-Vaccination-Informationsource | 302 | *“But because of the great pain I was in, and because my cousin on my mother's side had to die from it in Syria, and also my cousin on my father's side, I decided to take the vaccine so that when I am affected again, the disease will be somewhat milder.”(female, 56y.)* |
| Knowledge | Correct knowledge, wrong information and need for information, divided into vaccination knowledge, prevention knowledge, virus knowledge | 206 | *“Of course, there are side effects with every vaccination, regardless of whether you are also vaccinated against measles, mumps or rubella. Something can always happen that you somehow don't feel so well for one or two days.”(female, 48 y.)* |
| **COVID Rules** |  | **343** |  |
| Generic rules | Compliance and non compliance to the rules and the reason for it | 194 | *“I try to stick to the rules 90 percent of the time. I also see that these masks, as annoying as they are on the one hand, it made a difference. We have now had no cold and no flu in the winter.”(female, 66y.)* |
| Behaviour with a COVID-infection | Compliance and non compliance to the rules and the reason for it | 81 | *“She called, they were sent for PCR test, it was positive, accordingly they were quarantined for 2 weeks. I don't remember exactly, but 2 weeks she was not at work, then you do the test again, if it is negative, you go to work.”(female, 46 y.)* |
| Behaviour after contact to an infected person | Compliance and non compliance to the rules and the reason for it | 35 | *“Some people know what to do. Others don't know because they think, "The other person I met with is the infected person, not me, so I don't care.” (female, 35y.)* |
| Resources | Already existing resources to comply with the rules like (free tests, masks, benefits of rules) | 7 | *“Well, there are masks, we have been given so many masks.”(female, 67y.)* |
| Fears | Fears regarding the COVID-rules | 11 | *“Or the mask many do not put on because they have panic attacks”(female, 66y.)* |
| Needs | Needs for complying with the COVID-rules , named by the interviewee. | 15 | *“So not only in one direction say yes, here three G rules and or ventilation or what do I know what? But also much more to get in touch with the people personally.”(female, 46y.)* |
| **COVID Vaccination** |  | **390** |  |
| Information source | Source of information about vaccination against coronavirus | 10 | *„Because the family doctor was an authority for her and when he said get vaccinated, she had it done.”(female, 66y.)* |
| Unwilling/negative attitude | Unwilling to get vaccination against coronavirus or having a negative attitude towards COVID-19 vaccination, reasons for it | 61 | *“Some say it's nothing serious. They just don't want to get vaccinated. Some don't even believe the corona exists to get vaccinated against it”(male, 39y.)* |
| Vaccination hesitancy | Still unsure about getting vaccinated, reasons for it | 22 | *“I haven't felt that confident with it yet, or I didn't know how safe it was. But I think I will just get vaccinated soon.” (female, 19y.)* |
| Vaccinated/positive attitude | Already vaccinated or positive towards it, also including people who are vaccinated but changed their mind afterwards. Including reasons for it. | 144 | *„And I think vaccination is good, that you can slowly get into normality. And lead normal life.“ (female, 48y.)* |
| Children/Family | Vaccination attitudes regarding the family and children of the interviewee (Unwilling/negative attitude, hesitancy, vaccinated/positive attitude) | 74 | *“As a father, why should I get the vaccination and my children not? They will be vaccinated.“(male, 39y.)* |
| Ressources | Already existing resources for vaccination (eg. Vaccination truck) | 22 | *„On Facebook, you just see what your friends share. Maybe they are so totally in favor of vaccinations and then they share that and then you see that on their page. Well, he's doing it too, maybe I should get vaccinated too. Something like that. Maybe it's also quite good.“ (male, 33y.)* |
| Needs | Needs for getting vaccinated against the coronavirus, named by the interviewee. | 22 | *“We need to talk, convince, of course, I think we need to explain to people that there are no cases where people have died from vaccination, such mass vaccination.”(female, 67y.)* |
| Concerns | Concerns regarding the COVID- vaccination | 35 | *„But if you now force a person to get vaccinated and then something happens, then of course you don't want to have that on you somehow, this guilt, this feeling of guilt that, okay, that you now have pushed a person to get vaccinated and then somehow something happens.”(female, 21y.)* |
| **Corona-Daily life** |  | **546** |  |
| Rapid test | Including all information on COVID-19-rapid tests, including test obligation, reasons for testing, reasons against testing | 15 | *„ for example, his father he cannot visit him without taking a Corona test.”(female, 61y.)* |
| No effect | Interviewees state that COVID-19 has no impact on their daily life or the life of their surrounding | 2 | *“But for me personally, not much has changed because I'm a person who likes to be out in nature anyway. So I'm not someone who often goes to entertainment centers et cetera. “ (male, 46y.)* |
| Neutral impact | Interviewees state that COVID-19 has an impact on their life or the life of their surrounding, but it is stated without rating | 8 | *„In the beginning, they were also extremely careful to disinfect everything, to disinfect their hands every 10 minutes, but in the meantime, just like me, they have become a bit more relaxed, you can say.”(female, 37y.)* |
| Positive impact | Interviewees state that COVID-19 has a positive impact on their life or the life of their surrounding | 16 | *„That's when the families realized how much they had actually drifted apart, because from one day to the next they had to be together again in the smallest of spaces.“(female, 46y.)* |
| Negative impact | Interviewees state that COVID-19 has a negative impact on their life or the life of their surrounding | 195 | *“So, people who live alone, I can already imagine that they are lonelier. For older citizens, who could not see their children and grandchildren for a long time, it was certainly not easy..”(female, 54y.)* |
| Needs | Needs for their daily life, named by the interviewee, including financial, social, material support, desire for normality, reasons for health mediators, accessibility to services, general education | 186 | *„That it's over as soon as possible and that no one gets sick.”(female, 46y.)* |
| Resources | Already existing resources for the daily life in the COVID-pandemic. Indcuding supporting services. Resources fort he vaccination will be coded under vaccination and resources for complying with COVID-rules is coded under COVID-rules | 68 | *“there is here from the GEWOBA, I know that there is also the possibility to make somewhere to a contact that helps you. If you want to get shopping help for old people or something similar. I say, you can see where society then stands together, I think that's great too.” (male, 57)* |
| Concerns | Concerns regarding the daily life during the COVID-pandemic, including fears, psychological impact, concerns regarding the wider society | 56 | *„It is the same with my acquaintances. They are also afraid that they can't go anywhere because of Corona. They can't party. They can't go to the disco. You can't go anywhere because everyone is afraid that they will catch it if they go there.”(female, 35y.)* |
