# Supplementary figures and images for: Involving trained community health mediators in COVID-19 prevention measures. A process evaluation from Bremen, Germany
Source: Front Digit Health. 2023 Oct 11;5:1266684. doi: 10.3389/fdgth.2023.1266684 (PMC10598750; doi:10.3389/fdgth.2023.1266684)

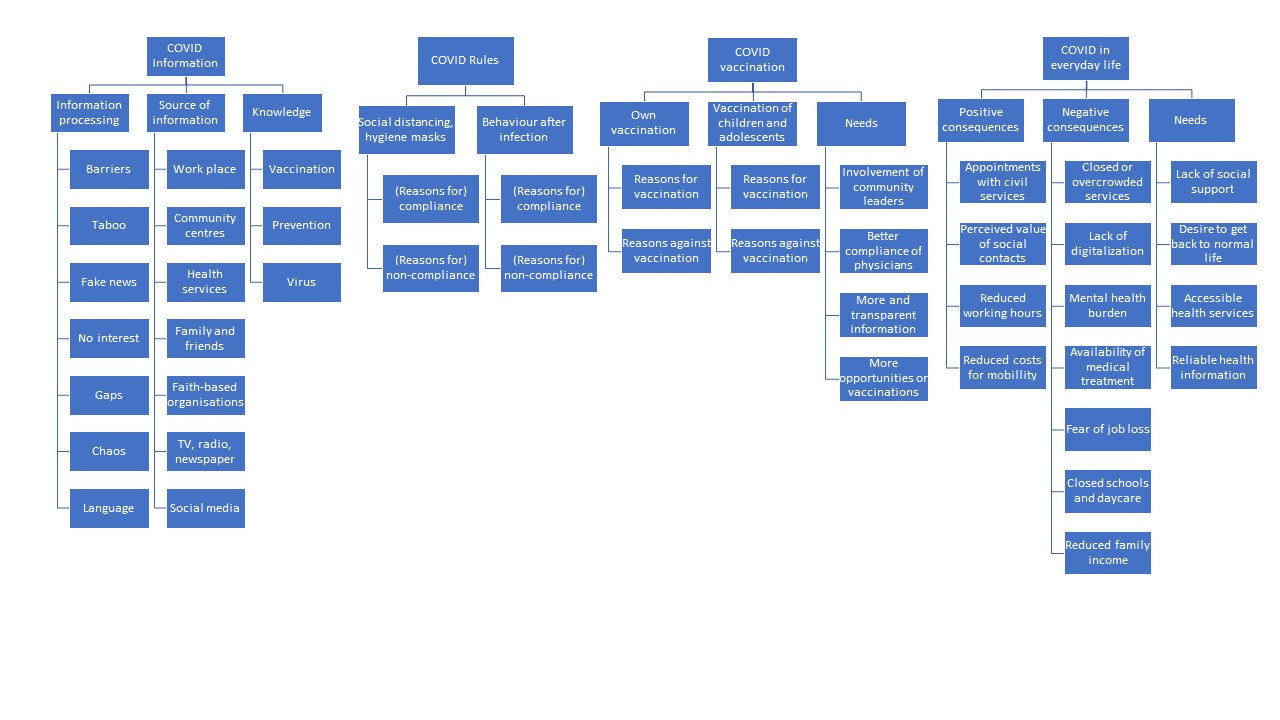

Supplement: Supplementary file 3 [file Image1.jpeg]
